# Intraoperative 3D transoesophageal valvular evaluation

**DOI:** 10.1186/1749-8090-8-S1-O79

**Published:** 2013-09-11

**Authors:** T Anguseva, I Milev, E Idoski, Z Mitrev

**Affiliations:** 1 Special Hospital for Surgery Fillip II, Skopje, Macedonia

## Background

The aims of this study were to evaluate the feasibility of real-time 3-dimensional (3D) transesophageal echocardiography in the intraoperative assessment of valvular pathology and to compare this novel technique with 2-dimensional (2D) transesophageal echocardiography.

## Methods

450 consecutive patients undergoing valvular were studied prospectively. Intraoperative 2D and 3D transesophageal echocardiographic (TEE) examinations were performed using a recently introduced TEE probe that provides real-time 3D imaging. Expert echocardiographers blinded to 2D TEE findings assessed the etiology of MR on 3D transesophageal echocardiography.

Similarly, experts blinded to 3D TEE findings assessed 2D TEE findings. Both were compared with the anatomic findings reported by the surgeon.

## Results

At the time of surgical inspection, ischemic MR was identified in 12% of patients, complex bileaflet myxomatous disease in 31%, and specific scallop disease in 25%, aortic stenosis in 20% and insufficiency in 12% of patients. Three-dimensional TEE image acquisition was performed in a short period of time (60-18 seconds) and was feasible in all patients, with optimal (36%)or good (33%) imaging quality in the majority of cases. Three-dimensional TEE imaging was superior to 2D TEE imaging in the diagnosis of P1, A2, A3, and bileaflet disease (P 0 .05), as well as in aortic stenosis and insufficiency evaluation (leaflet morphology).

## Conclusions

Real-time 3D transesophageal echocardiography is a feasible method for identifying specific valvular pathology in the setting of complex disease and can be expeditiously used in the intraoperative evaluation of patients undergoing valvular repair surgery.

